# The expression and clinical relevance of PD-1, PD-L1, and TP63 in patients with diffuse large B-cell lymphoma

**DOI:** 10.1097/MD.0000000000006398

**Published:** 2017-04-14

**Authors:** Xia Fang, Bing Xiu, Zhizhang Yang, Weizhe Qiu, Long Zhang, Suxia Zhang, Yunjin Wu, Xuyou Zhu, Xue Chen, Suhong Xie, Xianghua Yi, Aibin Liang, Yu Zeng

**Affiliations:** a Department of Hematology; b Deparment of Pathology, Tongji Hospital, Tongji University School of Medicine, Shanghai, China; c Division of Hematology and Internal Medicine, Mayo Clinic, Rochester, MN; d Department of Laboratory, Shanghai Zhongliu Hospital, Shanghai Fudan University School of Medicine, Shanghai, China.

**Keywords:** diffuse large B-cell lymphoma, programmed cell death 1, programmed cell death ligand 1, TP63

## Abstract

Latest study showed that a novel translocation between programmed cell death ligand 1 (PD-L1) (cluster of differentiation 274) and TP63 (tumor protein 63) can be found in diffuse large B-cell lymphoma (DLBCL), resulting in their conjunct overexpression in tumor cells at RNA level. However, the expressed pattern of these 2 genes at protein level in DLBCL remains largely unknown, and the clinical relevance of PD-L1 and TP63 expression in DLBCL are also unclear.

Tumor tissues from 76 Chinese DLBCL patients were immunostained for programmed cell death 1 (PD-1), PD-L1, and TP63 using the EnVision system. Clinical relevance of PD-1, PD-L1, and TP63 in 74 DLBCL were analyzed by chi-square test, the Kaplan–Meier curves with log rank test, and Cox's proportional hazards regression model.

PD-1 was mainly expressed in tumor-infiltrating lymphocytes (TILs) of 39.5% patients. PD-L1 was expressed in tumor cells of 26.3% patients, and TP63 was immunostained in nucleoli of tumor cells of 31.6% cases. PD-1 expression was significantly associated with the patients’ gender and B symptoms (*P* = 0.032, *P* = 0.026). DLBCL with PD-L1 or TP63 expression in tumor cells showed low International Prognostic Index (IPI) score (*P* = 0.007, *P* = 0.009). PD-1^+^ TILs was related to prolonged overall survival rate (OS) of DLBCL patients (*P* = 0.02), whereas PD-L1 expression was associated with worse clinical outcome of patients (*P* = 0.049). Immunoreactivity of TP63 was not correlated with patients’ survival time. Besides, PD-1 expression, patients’ age, Ann Arbor stage, and IPI score were significant prognostic markers for OS, but PD-L1 and TP63 had no prognostic significance.

PD-1, PD-L1, and TP63 are frequently expressed in DLBCL. PD-1/PD-L1/TP63 blockade may be a potential therapeutic strategy for some patients.

## Abstract 2

Supplemental Digital Content is available in the text

## 1 Introduction

Programmed cell death ligand 1 (PD-L1, also known as CD274 or B7H1), a member of the B7 receptor family, is an inhibitory receptor expressed on activated T cells, B cells, dendritic cells, macrophages, and natural killer T (NKT) cells.^[1,2]^ PD-L1 delivers a co-inhibitory signal upon binding to its cognate receptor PD-1, which suppresses proliferation of activated T cells in peripheral tissues.^[3]^ Recently, PD-1/PD-L1 blockade enhance anti-tumor immune response in many solid tumors, such as non-small cell lung cancer (NSCLC), melanoma, and renal cell carcinoma.^[4–9]^ Therefore, targeting the PD-1/PD-L1 axis may be a potential utility in hematologic malignancies.

In previous clinical trials, PD-1 and PD-L1 were found to be aberrantly expressed in variety of lymphoma, including T-cell lymphoma, mediastinal large B-cell lymphoma, classical Hodgkin lymphoma (cHL), and anaplastic large cell lymphoma.^[10–13]^ The blockade PD-1/PD-L1 pathway demonstrated beneficial therapeutic effect in cHL and follicular lymphoma.^[14,15]^ In diffuse large B-cell lymphoma (DLBCL), PD-1 was expressed on tumor-infiltrating lymphocytes (TILs), and the number of PD-1^+^ TILs was associated with the favorable overall survival (OS) in patients with DLBCL.^[16,17]^ Besides, the higher expression of PD-L1 was also found in tumor cells and it maybe a potential biomarker for DLBCL.^[18]^ Thus, the PD-1/PD-L1 pathway may also contribute to the proliferation of tumor cells of DLBCL and may be a novel applicable strategy to treat patients with DLBCL.

Lately, some effort put on clarifying why PD-1 or PD-L1 is frequently expressed in some hematologic malignant cells. The structural variation frequently disrupting the 3′ region of PD-L1 was identified in 27% adult T-cell leukemia/lymphoma and in 8% DLBCL, which resulted in the arrangement of gene's open reading frame.^[19]^ Similarly, the PD-L1 and TP63 fusion translocation pattern was newly found in 5% (1/20) DLBCL, leading to jointly increasing the mRNA expression level of both genes.^[20]^ Based on this intriguing finding, in this study we investigated the expression of PD-L1 and TP63 in 76 DLBCL cases by immunohistochemistry, and studied the frequency of their conjunct aberrant expressions in DLBCL. The present study aims to identify whether PD-L1 upregulation accompanies with the abnormal expression of TP63 in DLBCL at protein level, and whether their expressions are significantly correlated with each other. Besides, our study also investigated the clinical implication of PD-L1/TP63 in patients with DLBCL. Importantly, our research wants to uncover that PD-L1/TP63 immunostaining may be a preliminary screening for further molecular detection of some DLBCL cases, and targeting PD-L1/TP63 may be an ideal treatment for some patients.

## 2 Method

### 2.1 Patients

A total of 76 patients diagnosed with DLBCL at Shanghai Tongji Hospital affiliated with Tongji University School of Medicine were enrolled into this study. All cases were reviewed according to present World Health Organization criteria (WHO 2008). Among these cases, 43 were treated with chemotherapy, 22 received surgery treatment, and 3 cases received chemotherapy after surgery treatment. The clinical data and outcomes were obtained from the patients’ medical records, including patients’ age, sex, grade, International Prognostic Index (IPI) score, and so forth. The follow-up period ranged from 0.2 to 7.2 years. Response criteria were based on standard guidelines. A total of 74 cases had clinical information and were included in survival analysis. This research was approved by the ethic committee of Tongji Hospital, Tongji University School of Medicine. The informed consents were obtained from all patients.

### 2.2 Histopathological and immunohistochemical analysis

For immunohistochemistry (IHC) analysis, 3-mm-thick formalin-fixed paraffin-embedded sections were submitted. All histological and immunophenotypic data were reviewed by 2 pathologists under blind study. DLBCL was classified into the GCB and non-germinal center B-cell (non-GCB) phenotype according to the Hans algorithm based on IHC for CD10, Bcl-6, and MUM1.The immunohistochemical staining for detection of PD-1, PD-L1, and TP63 were performed using EnVision system, the detail process was described as the previous study.^[21]^ The information of antibodies was used for IHC detection as following: monoclonal mouse anti-human-CD10 (Clone 56C6; Dako, 1:100 dilution), monoclonal mouse anti-human-Bcl6 (Clone PG-B6p; Dako, 1:10 dilution), monoclonal mouse anti-human-MUM1 (Clone MUM1p; Dako, 1:50 dilution), monoclonal mouse anti-human-PD-1(MRQ-22; ZSGB-BIO, ready to use), monoclonal rabbit anti-human PD-L1 (SP142;ZSGB-BIO, ready to use), and monoclonal mouse anti-human-TP63 (DAK-p63; Dako, 1:50 dilution).

The expression of PD-L1 was evaluated as the previous study.^[22]^ The proportion of the cells showing membranous and/or cytoplasmic staining was assessed as follows: no or any staining fewer than 10% of tumor cells are negative and staining of more than 10% of tumor cells are positive. The intensity of positive cells was scored as follows: 1, weak; 2, moderate; and 3, strong staining. The proportion of immunostained cells was evaluated among tumor cells and tumor-infiltrating immune cells, respectively. The number of PD-1^+^ cells in TILs was assessed semi-quantitatively and scored as the previous study^[22]^:0, no positive cells/high-power field; 1, fewer than 10 positive cells/high-power field; 2, 10 to 30 positive cells/high-power field; 3, more than 30 positive cells/high-power field on average. We used a cut-off value of 30% immunoreactivity for TP63 based on the previous study.^[23,24]^ The 10% cut-off value of PD-L1 expression and 30% cut-off value of TP63 expression were adopted to identify cases as positive or negative for these 2 proteins. Each case was randomly selected at least 5 fields and quantified.

### 2.3 Statistical analysis

The statistical analysis was performed using SPSS 16.0 (SPSS Inc., Chicago, IL). The clinical relevance of PD-1, PD-L1, and p63 in DLBCL was assessed using the *χ*^2^ test (N >5) or Fisher's exact test (N). Two-sided *P* values were calculated and *P* <0.05 was considered significant. The Kaplan–Meier curves with log rank test were employed for the survival analysis. The correlations between PD-L1 and TP63 expression were evaluated using Pearson correlation and linear regression analysis. The Cox-regression analyses, both univariate and multivariate, were used to identify the independency of these genes expression status. Both hazards ratio and 95% confidence intervals were calculated for the univariate and multivariate analyses of OS, and *P* <0.05 was considered significant.

## 3 Results

### 3.1 Clinicopathological characteristics of patients with DLBCL

The clinicopathological features of patients with DLBCL are shown in Table 1. Briefly, the median age of patients was 63 years (range: 25–92), with 55.4% of male and 44.6% of female. 22.9% patients were diagnosed with GCB-type DLBCL, whereas 77.1% patients were diagnosed with non-GCB-type DLBCL. Among these cases, primary extranodal disease (47/74, 63.5%) and low IPI score 0 to 2 (56/74, 75.6%) accounted for the large proportion of patients. Epstein–Barr virus (EBV) infection was observed in 2.7% of the patients. Patients who accepted R-CHOP or R-CHOP-like treatment accounted for 58.1%, and patients who received surgery therapy were 29.7%.

**Table 1:**
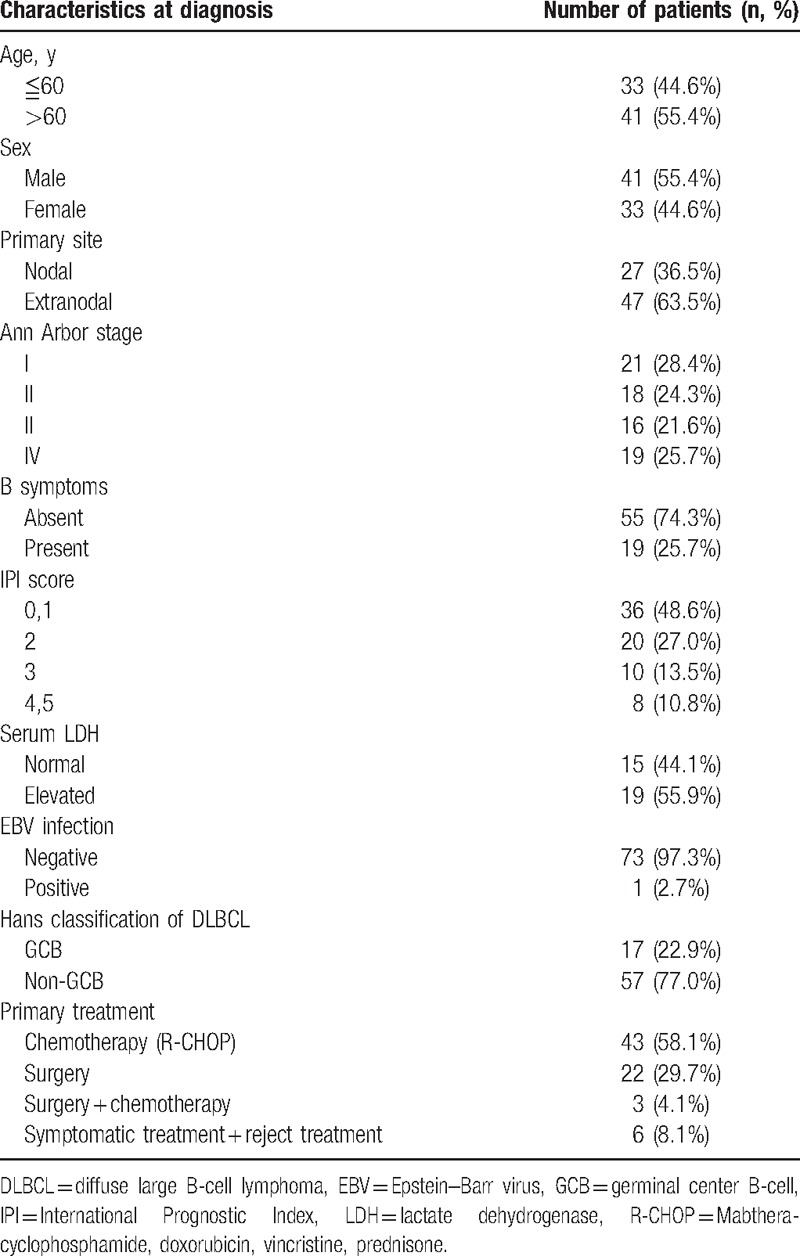
Clinical information of patients with DLBCL.

### 3.2 The expression of PD-1, PD-L1, and TP63 in DLBCL tissues

**Figure 1:**
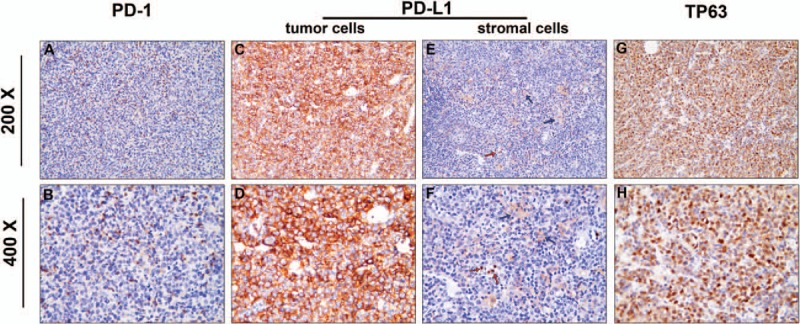
Representative images for PD-1, PD-L1, and TP63 expressions in DLBCL (upper lane: 200×, lower lane: 400×; EnVision). (A and B) PD-1 was immunostained in TILs. c–f: Immunostaining for PD-L1 showed that it was mainly expressed in the membrane and/or cytoplasm of tumor cells (C and D) and in the stromal cells mainly macrophages (blue arrow) and neutrophils (red arrow) (E and F). (G-H) Immunostaining for TP63 demonstrated that it was expressed in the nucleoli of tumor cells. DLBCL = diffuse large B-cell lymphoma, PD-1 = programmed cell death 1, PD-L1 = programmed cell death ligand 1, TIL = tumor-infiltrating lymphocytes, TP63 = tumor protein 63.

The expression of PD-L1^+^, TP63^+^ in malignant cells, and PD-1^+^ in tumor-infiltrating cells was summarized in Supplemental Table S1. Overall, PD-1 was mainly stained in the TIL of 39.5% patients (Supplemental Table S1, Fig. 1A and B). Immunostaining for PD-L1 in representative cases showed that it was expressed in the membrane and/or cytoplasm of tumor cells (Fig. 1C and D). In our cases, 26.3% patients possess PD-L1 expression with an IHC score of 1 in 6.6% patients, 2 in 13.1%, and 3 in 6.6% of evaluated cases. PD-L1 staining was also observed in the macrophages and neutrophils (Fig. 1E and F). 31.6% patients showed TP63 immunoreactivity in the nucleoli of tumor cells with 1.3% case of weak positive, 13.1% of moderate, and 17.2% of strong positive (Supplemental Table S1, Fig. 1G and H). To investigate whether PD-L1 and TP63 were conjunctly expressed in DLBCL patients, we further analyzed their double-positive cases. The results showed that 11 cases (11/76, 14.5%) were double immunoreactivity of PD-L1 and TP63 with 10 non-GCB-type and 1 GCB-type DLBCL (Supplemental Table S1). Additionally, we found that there was a positive correlation between PD-L1 and TP63 expression (*P* = 0.032, *r* = 0.0247), although the relevancy is not quite obvious.

### 3.3 Clinicopathological analysis and prognostic significance of PD-1, PD-L1, and TP63 expressions in DLBCL

The clinical implications of PD-1, PD-L1, and TP63 in patients with DLBCL are summarized in Table 2. Briefly, PD-1 expression in TILs was significantly higher in male patients and patients without B symptoms (*P* = 0.032, *P* = 0.026, respectively, Table 2). Patients with PD-L1 or TP63 expression were more likely to have lower IPI score (*P* = 0.007, *P* = 0.002, respectively, Table 2). This may be because of more patients with low IPI score in our study. Besides, TP63 expression in tumor cells was related to Ann Arbor stage (*P* = 0.031, Table 2). There were no significant correlation between other clinical variables and these proteins’ expression (Table 2).

**Table 2:**
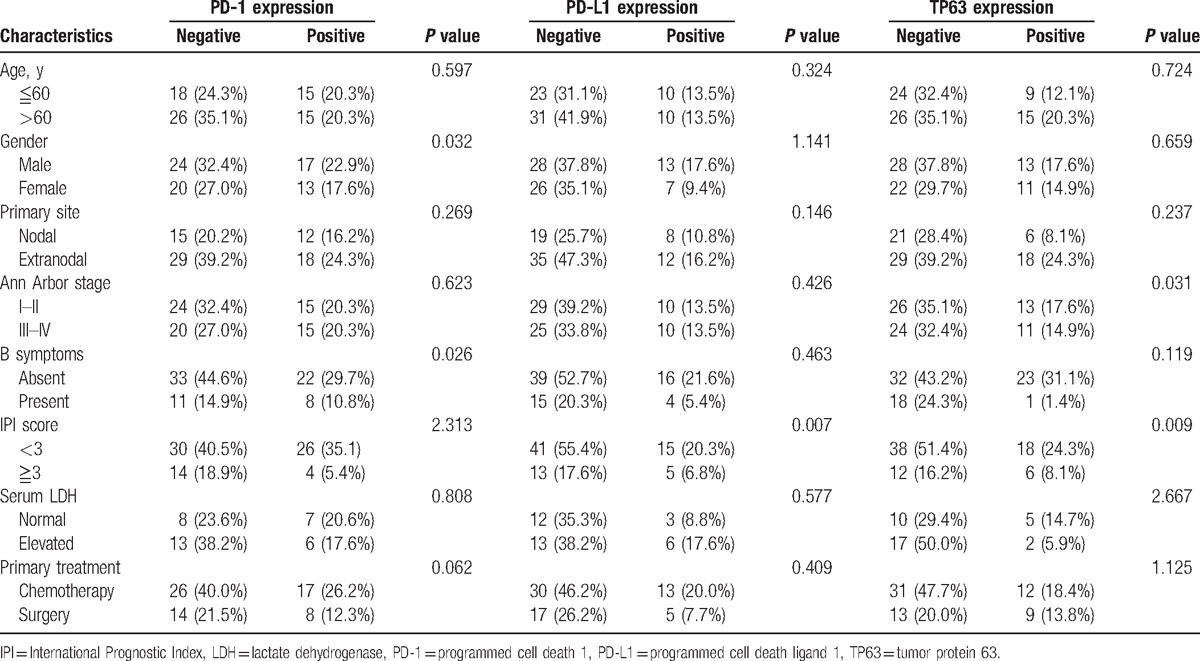
Clinical relevance of PD-1, PD-L1, and TP63 expressions in DLBCL.

Additionally, we investigated the clinical relevance of these genes in patients who only received the chemotherapy treatment. The results showed that PD-1 expression in TILs was significantly higher in male patients with low IPI score (*P* = 0.002, *P* = 0.000, respectively, Supplemental Table S2). Similarly, patients with PD-L1 and TP63 expressions possess low IPI score and without B symptoms (Supplemental Table S2).

**Figure 2:**
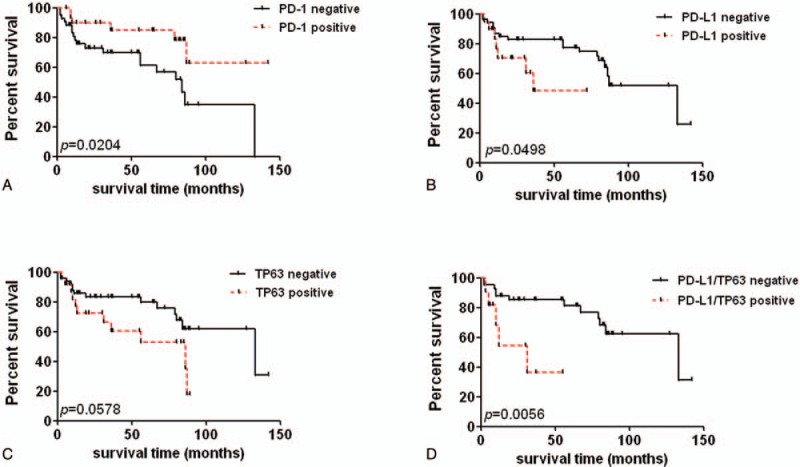
Comparison of survival rates according to PD-1, PD-L1, and TP63 expression. (A) The survival rate of DLBCL patients with PD-1 negative and PD-1 positive expression (*P* = 0.02). (B) The correlation between PD-L1 expression and patients’ overall survival rate (*P* = 0.0498). (C) The survival rate of TP63 negative and positive cases (*P* = 0.0578). (D) Double-positive expression of PD-L1 and TP63 is associated with worse clinical outcome of DLBCL (*P* = 0.0056). DLBCL = diffuse large B-cell lymphoma, PD-1 = programmed cell death 1, PD-L1 = programmed cell death ligand 1, TP63 = tumor protein 63.

Kaplan–Meier analysis revealed that patients with PD-1 expression in TILs had prolonged survival time compared with PD-1-negative cases (Fig. 2A, *P* = 0.02). Whereas, PD-L1 expression in tumor cells possessed worse OS rate (Fig. 2B, *P* = 0.0498). Furthermore, TP63 positive cases were not associated with OS in DLBCL patients (Fig. 2C, *P* >0.05). Notably, patients with double-positive expression of PD-L1 and TP63 were significantly had worse clinical outcome compared with those with PD-L1/TP63 double negative (Fig. 2D, *P* = 0.005). In chemotherapy-treated patients, Kaplan–Meier analysis revealed that PD-L1 expression in tumor cells was significantly related to patients’ poor survival rate (Supplemental Fig. S1*P* = 0.041), but patients with double positive of PD-L1 or TP63 expression were not associated with patient's survival rate.

Univariate analysis showed that OS was associated with patients’ age, Ann Arbor stage, IPI score, and PD-1 expression (Table 2, *P* = 0.007, *P* = 0.029, *P* = 0.000, *P* = 0.036, respectively). Multivariate analysis was performed to identify whether they are independent prognostic marker for OS, and the result revealed that age, Ann Arbor stage, IPI score, and PD-1 score are independent prognostic factor for OS (Table 3). Besides, univariate analysis by the Cox hazard model revealed that PD-L1 was not an independent prognostic factor for patients’ OS, although its expression was associated with worse survival time of DLBCL patients (Table 3, *P* >0.05). In only chemotherapy cohort, univariate analysis revealed that PD-1, PD-L1, and TP63 expressions were not correlated with patients’ age, gender, B symptoms, Ann Arbor stage, and IPI score maybe because of the relatively small sample size.

**Table 3:**
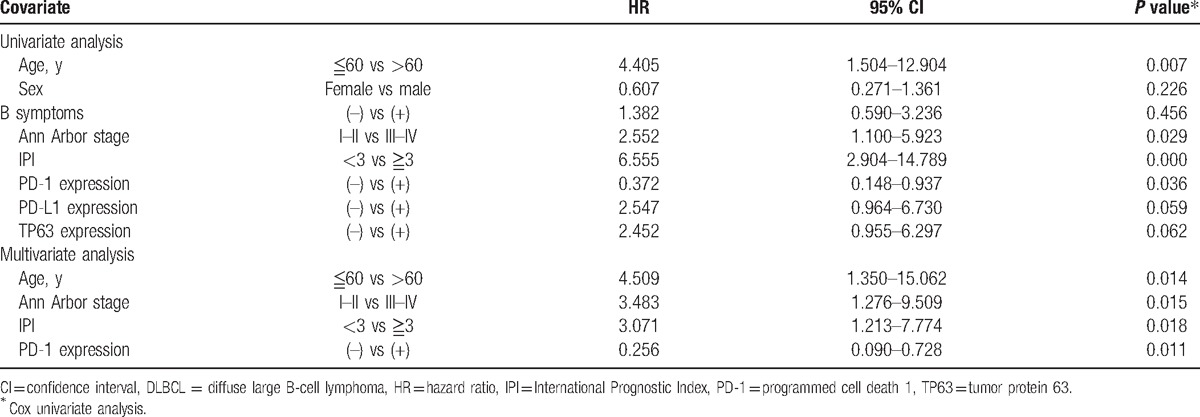
Univariate and multivariate analysis of PD-1, PD-L1, and TP63 expressions and overall survival of DLBCL.

## 4 Discussion

In the present study, high expressions of PD-1, PD-L1, and TP63 were found in some patients with DLBCL. Patients with positive of PD-1 and PD-L1 were significantly associated with patients’ survival rate. The univariate analysis showed that OS rate was related to patients’ age, Ann Arbor stage, IPI score, and PD-1 expression, and the multivariate analysis further validated that these factors are independent prognostic markers for patients’ OS. Overall, our study suggests that PD-L1 or PD-1 maybe a potential biomarker or treated target for some DLBCL patients.

Previous studies indicated that PD-1 and PD-L1 expressions in tumor cells or immune cells is meaningfully associated with the patients’ response to PD-1 /PD-L1 blockade.^[25,26]^ All the time, NSCLC was identified with no-responsiveness to immunotherapy until the high expression of PD-1/PD-L1 was found in tumor cells or immune cells.^[27,28]^ Thus, the PD-1 monoclonal antibody (nicolumab) exhibited objective responses in cancer patients with high expression of PD-1/PD-L1 in contrast with PD-1/PD-L1-negative patients.^[29,30]^ Evaluating PD-1 expression on immune cells or PD-L1 expression on tumor cells and immune cells may be valuable for predicting responsiveness to PD-1/PD-L1 immunotherapy in malignant tumors. In our cases, PD-1 was highly expressed on immune cells and PD-L1 was highly expressed in tumor cells and immune cells of some cases, indicating that the PD-1/PD-L1 axis may also be useful for some patients with DLBCL. To further analyze the clinical significance of PD-1 and PD-L1 in our cases, we found that DLBCL patients with PD-1^+^ TILs had prolonged survival time, which contradicts with the suppressed role of PD-1 in immune response. However, the increased PD-1^+^ cells might reflect previous active immune response as Dohee's described, thus PD-1 expression was associated with favorable survival rate of patients.^[23]^ Consistently, the multivariate analysis by the Cox hazard model showed that PD-1 is an independent prognostic marker for patients’ OS rate. Being consistent with Kiyasu and colleagues’^[31]^ study, our data also revealed that PD-L1 expression was correlated with worse clinical outcome although it is not an independent prognostic marker for patients’ OS in our study, which suggested that treatments targeting PD-L1 might benefit DLBCL patients.

Additionally, only a chemotherapy-treated cohort can be used to investigate the clinical relevance of PD-1 and PD-L1. Consistently, PD-L1 is significantly associated with patients’ survival rate, but PD-1 is not related to patient's survival rate. The multivariate analysis by the Cox hazard model displayed that all of them are not independent prognostic marker for patients’ OS rate. Therefore, more samples may be needed for further studies to validate the clinical application of them in DLBCL.

Recently, some studies indicate that the high expression of PD-L1 can be induced by many oncogene or genetic aberrations. Loss function of PTEN was reported to up-regulation PD-L1 expression in tumor cells.^[32]^ The structural variation frequently disrupting the 3′ region of PD-L1's open reading frame, which leads to its overexpression.^[19]^ Interestingly, Georgiou and colleagues^[20]^ discovered that a novel translocation juxtaposing between PD-L1 and TP63 in DLBC, and RNA-seq data also showed that the high expression of PD-L1 is accompanied with TP63 expression. It is well known that TP63 is critical for cell homeostasis and embryo and epidermal development.^[33,34]^ Several studies showed the aberrant expression and structural abnormality of TP63 was found in multiple types of lymphoma.^[35,36]^ Moreover, Fukushima et al^[24]^ uncovered that TP63 was expressed in the nucleoli of tumor cells in 34% DLBCL, and non-germinal center DLBCL with TP63 positive displayed worse prognosis. In the present study, TP63 expression was found in 32.9% patients. Additionally, since previous study showed that PD-L1 may accompanied with TP63 expression,^[20]^ thus we wonder whether their expression was associated with each other, and whether they are prognostic marker for DLBCL patients. Our results demonstrated that TP63 positive expression was associated with PD-L1 expression though the significance was not obvious perhaps because of small sample size. Besides, our results also showed that patients with double positive of PD-L1 and TP63 had worse survival. A recent study indicated that the AKT/mTOR signaling pathway could be directly activated by PD-1/PD-L1 during the malignant progression of DLBCL.^[36]^ Similarly, TP63 also could regulate mTOR signaling, which play a critical role in the progress of DNA damage.^[37]^ Our previous study showed that PD-L1 dysfunction could promote the progression of leukemia via regulating JNK/cyclin D2 signaling.^[38]^ Thus, we speculated that PD-L1/TP63's overexpression may activate some important intracellular signaling results in tumor development, which need more further studies to elucidate. However, in only chemotherapy-treated cohort, no significance was to be found in correlation between PD-L1 and TP63 expression as well as the correlation between double positive of PD-L1/TP63 and patients’ survival rate. Therefore, more sample size and further studies need to clarify whether PD-L1 and TP63 have synergistic effect and whether the traslocational fusion of them play a critical role in the progression of DLBCL.

In summary, our study uncovers that PD-1, PD-L1, and TP63 are highly expressed in some DLBCL. PD-1 predicts favorite patients’ survival rate and is an independent prognostic marker of DLBCL. PD-L1 is associated with patients’ poor prognosis regardless of the whole cohort and only chemotherapy-treated cohort. However, PD-L1 and TP63 show weak correlation in all patients, and no significance was found in the only chemotherapy-treated cohort. Additionally, double positive of TP63 and PD-L1 is not related to patients’ OS rate in only chemotherapy-treated patients, but their expression is associated with the OS rate in all patients. Thus, further studies are needed to clarify the expression pattern of PD-L1/TP63 and the role of PD-L1/TP63 translocational fusion in DLBCL by a large sample size.

## Supplementary Material

Supplemental Digital Content
