# Supplementary material for: The expression and clinical relevance of PD-1, PD-L1, and TP63 in patients with diffuse large B-cell lymphoma
Source: Medicine (Baltimore). 2017 Apr 14;96(15):e6398. doi: 10.1097/MD.0000000000006398 (PMC5403068; doi:10.1097/MD.0000000000006398)
Supplement: Supplemental Digital Content [file medi-96-e6398-s001.doc]

**sTable 1 The expression of PD-1, PD-L1 and TP63 in DLBCL**

| **Proteins** | **N (%)** |
| --- | --- |
| **PD-1^+^** | **30 (30/76, 39.5%)** |
| **PD-L1^+^** | **20 (20/76, 26.3%)** |
| **TP63^+^** | **24 (24/76, 31.6%)** |
| **PD-1^-^/PD-L1^+^** | **17 (17/76, 22.4%)** |
| **PD-1^+^/PD-L1^-^** | **28** **(28/76, 36.8%)** |
| **PD-1^-^/PD-L1^-^** | **28 (28/76, 36.8%)** |
| **PD-1^+^/PD-L1^+^** | **3 (3/76, 3.9%)** |
| **PD-1^-^/TP63^+^** | **16 (16/76, 21.1%)** |
| **PD-1^+^/TP63^-^** | **22 (22/76, 28.9%)** |
| **PD-1^-^/TP63^-^** | **29 (29/76, 38.2%)** |
| **PD-1^+^/TP63^+^** | **9 (9/76, 11.8%)** |
| **PD-L1^-^/TP63^+^** | **14 (14/76, 18.4%)** |
| **PD-L1^+^/TP63^-^** | **9 (9/76, 11.8%)** |
| **PD-L1^-^/TP63^-^** | **42 (42/76, 55.3%)** |
| **PD-L1^+^/TP63^+^** | **11 (11/76, 14.5%)** |

| **Characteristics**  **Negative Positive** | **PD-1 expression** | | ***P***  **value**  **Negative Positive** | **PD-L1 expression** | ***P***  **value**  **Negative Positive** | **TP63 expression** | | ***P***  **Value** |
| --- | --- | --- | --- | --- | --- | --- | --- | --- |
| **Age (year)**  **15 (34.8%)**  **≦60**  **11 (25.5%)**  **> 60** | **9 (20.9%)**  **8 (18.6%)** | **16 (37.2%)**  **13 (30.2%)**  **0.445** | | **8 (18.6%)**  **6 (13.9%)** | **0.120**  **18 (41.8%)**  **12 (27.9%)** | | **6 (13.9%)**  **7 (16.2%)** | **0.724**  **0.038** |
| **Gender**  **14 (32.5%)**  **Male**  **12 (27.9%)**  **Female** | **5 (11.6%)**  **12 (27.9%)** | **11 (25.6%)**  **18 (41.9%)**  **0.002** | | **8 (18.6%)**  **6 (13.9%)** | **1.141**  **19 (44.1%)**  **11 (25.5%)** | | **6 (13.9%)**  **7 (16.2%)** | **0.65**  **0.021** |
| **Priamry site**  **10 (23.2%)**  **Nodal**  **17 (65.4%)**  **Extranodal** | **7 (16.2%)**  **9 (34.6%)** | **18 (41.9%)**  **11 (25.6%)**  **0.189** | | **8 (18.6%)**  **6 (13.9%)** | **0.053**  **14 (32.5%)**  **16 (37.2%)** | | **3 (6.9%)**  **10 (23.2%)**  **0.027** |  |
| **Ann Arbor stage**  **14 (32.5%)** | **3 (6.97%)** | **0.267** | |  | **0.127** | |  | **0.599** |
| **Ⅰ-Ⅱ**  **12 (27.9%)**  **Ⅲ-Ⅳ**  **B symptoms**  **18 (41.8%)** | **14 (32.5%)**  **11 (25.5%)** | **12 (27.9%)**  **17 (39.5%)**  **19(44.1%)**  **0.096** | | **5 (11.6%)**  **9 (20.9%)**  **10 (23.2%)** | **0.004**  **13 (30.2%)**  **17 (39.5%)**  **17 (39.5%)** | | **4 (9.30%)**  **9 (20.9%)**  **12 (27.9%)** | **0.004** |
| **Absent**  **8 (18.6%)**  **Present** | **6 (13.9%)** | **10 (23.2%)** | | **4 (9.3%)** | **13 (30.2%)** | | **1 (2.3%)** |  |
| **IPI score**  **20 (46.5%)** | **15 (34.8%)** | **25 (58.1%)**  **0.000** | | **10 (23.2%)** | **26 (60.4%)**  **0.002** | | **9 (20.9%)** | **0.001** |
| **< 3**  **6 (13.9%)**  **≧3**  **Serum LDH**  **8 (33.3%)** | **2 (4.6%)**  **5 (20.8%)** | **4 (9.3%)**  **10 (41.6%)**  **0.002** | | **4 (9.3%)**  **3 (12.5%)** | **0.172**  **4 (9.3%)**  **8 (75%)** | | **4 (9.3%)**  **5 (20.8%)** | **0.053** |
| **Normal**  **8 (33.3%)**  **Elevated** | **3 (12.5%)** | **7 (29.1%)** | | **4 (16.6%)** | **10 (41.6%)** | | **1 (4.1%)** |  |

**sTable 2 Clinical relevance of PD-1, PD-L1 and TP63 expression in chemotherapy-treated DLBCL**

**
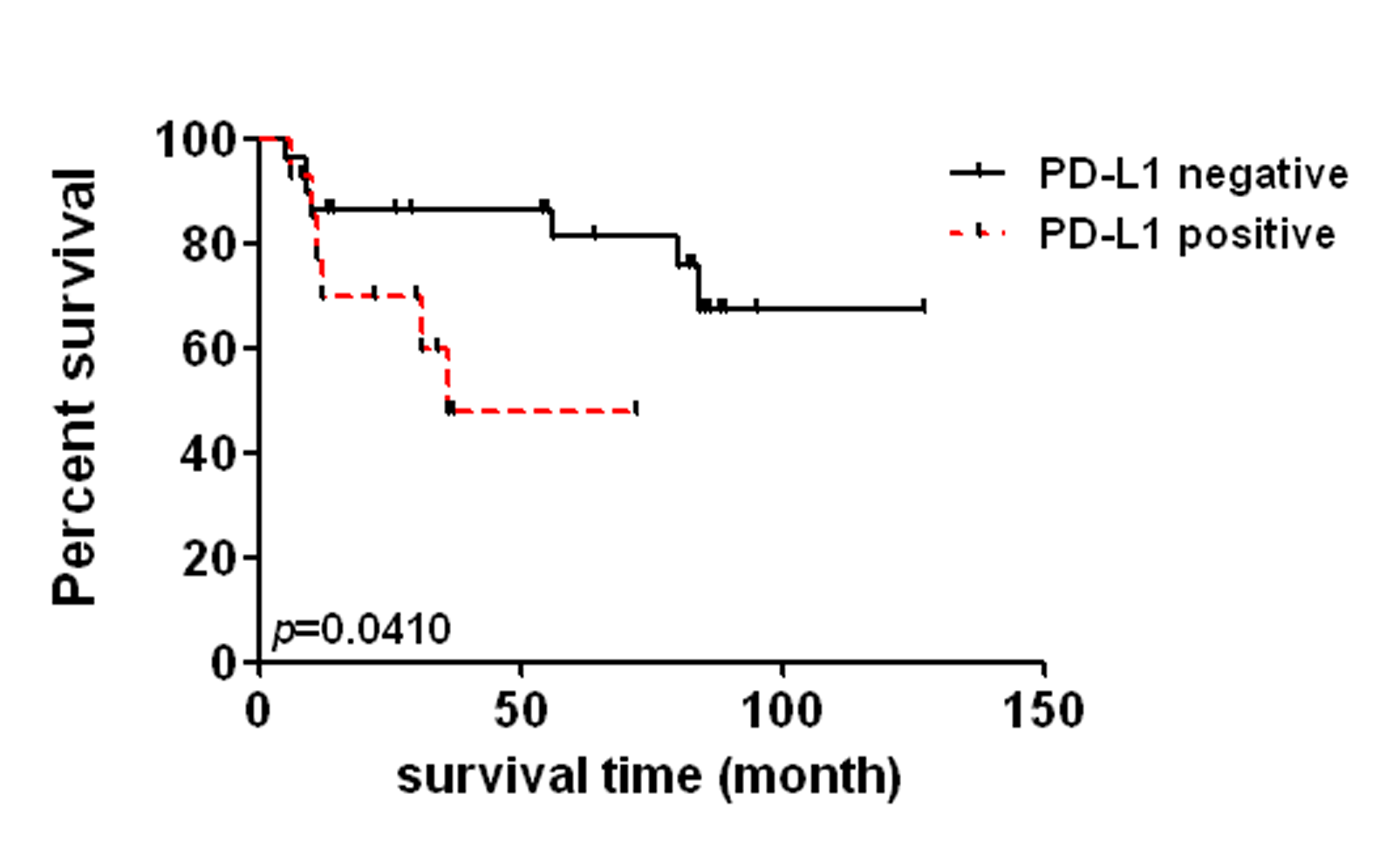
**

**sFigure 1 The survival rate of chemotherapy-treated patients according to PD-L1 expression.** The survival rate of chemotherapy-treated patients with PD-L1 negative and PD-1 positive expression (*p*=0.0410).
